# Influence of Gel-Type Confinement for Laser Shock Peening of a Ni-Based Alloy

**DOI:** 10.3390/ma18092145

**Published:** 2025-05-06

**Authors:** Sebastian Balos, Milan Pecanac, Dragan Rajnovic, Petar Janjatovic, Miroslav Dramicanin, Sanja Kojic, Filip Cap, Lidija Krstanovic, Sanin Zulic

**Affiliations:** 1 Department of Production Engineering, Faculty of Technical Sciences, University of Novi Sad, Trg Dositeja Obradovica 6, 21000 Novi Sad, Serbia; sebab@uns.ac.rs (S.B.); pecanac.milan@uns.ac.rs (M.P.); draganr@uns.ac.rs (D.R.); janjatovic@uns.ac.rs (P.J.); 2 Department of Energy, Electronics and Telecommunications, Faculty of Technical Sciences, University of Novi Sad, Trg Dositeja Obradovica 6, 21000 Novi Sad, Serbia; sanjakojic@uns.ac.rs; 3 Hilase Centre, Institute of Physics of the Czech Academy of Sciences, Za Radnicí 828, 252 41 Dolní Břežany, Czech Republic; filip.cap@hilase.cz (F.C.); sanin.zulic@hilase.cz (S.Z.); 4 Department of Fundamentals Sciences, Faculty of Technical Sciences, University of Novi Sad, Trg Dositeja Obradovica 6, 21000 Novi Sad, Serbia; lidijakrstanovic@uns.ac.rs

**Keywords:** laser shock peening, gel confinement, residual stresses, microhardness

## Abstract

Laser shock peening (LSP) significantly enhances fatigue and corrosion resistance, especially in additively manufactured components. This effect is stronger when confinement is used; typically, it is water. However, water poses risks to sensitive electronics. As an alternative, this study explored gel-based confinement. A Ni-based alloy was LSP-treated using 532 nm and 1064 nm wavelengths, with three types of gel compared to water as a control. The results showed that gel confinement can induce compressive residual stresses and increase surface microhardness. However, gels were generally less effective than water in terms of residual stress magnitude and depth of hardening. Additionally, gel confinement required the use of a 1064 nm laser, whereas water confinement was more effective with 532 nm. Among the gels tested, one adhesive variant performed best due to improved surface contact and strong adhesion. The observed increase in microhardness and compressive stress was linked to surface grain refinement and twinning. Overall, adhesive gels offer potential benefits for LSP, particularly for additively manufactured parts, which often have high surface roughness and require non-conductive confinement solutions.

## 1. Introduction

Laser shock peening (LSP) is an emerging surface treatment technology that induces beneficial compressive residual stresses (CRS) in the treated material. It is similar in effect to the more conventional shot Peening (SP), but LSP offers significantly higher strain rates and deeper penetration of the stress field, resulting in more pronounced industrial benefits [1]. Due to its versatility and effectiveness on metallic materials and alloys, LSP is now widely applied in advanced manufacturing across the aerospace [2], nuclear [3], transportation [4], and biomedical industries [5].

LSP operates by irradiating the material surface with repeated high-energy laser pulses [6]. This leads to rapid vaporization near the surface and intense ionization of atoms, generating a rapidly expanding plasma [7]. The plasma induces a high-pressure shock wave that propagates into the material, causing localized plastic deformation when the Hugoniot elastic limit (HEL) is exceeded [8]. The primary benefit of LSP is the induction of deep, stable compressive residual stresses [9].

**Figure 1:**
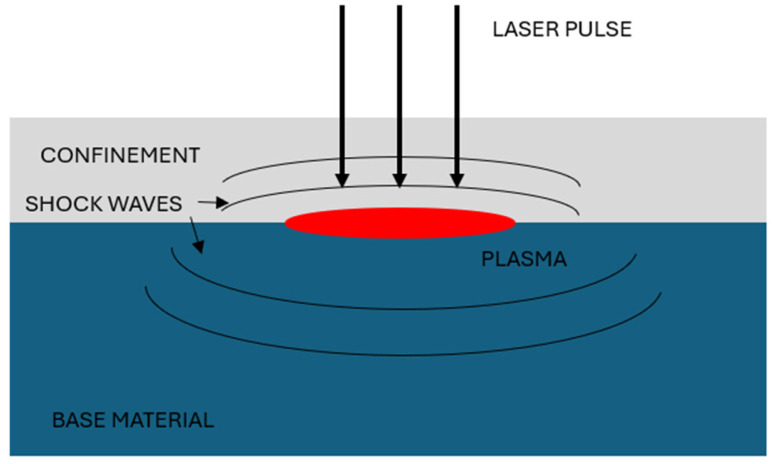
Schematic of the LSP process on the base material.

The key parameters affecting LSP performance include laser pulse wavelength, duration, shape, and energy density [10,11]. An important enhancement technique is the use of confinement, which significantly increases the shock intensity and effectiveness. Confinement results in more concentrated stress waves, improving fatigue life, corrosion resistance, surface hardness, wear resistance, and even tensile strength of the treated components [12,13,14,15,16,17]. A schematic of the LSP process with confinement is shown in Figure 1.

Water is the traditional confinement medium in LSP due to its abundance, low cost, and proven effectiveness under certain conditions [18,19]. However, practical challenges and limitations associated with water—such as its incompatibility with sensitive components—have led to the development of alternative confinement methods. One of the most prominent alternatives is solid confinement, typically in the form of glass [20,21] or polymers [22,23].

Solid confinements offer specific advantages over water, particularly in hybrid processes such as laser powder bed fusion (LPBF), where they contribute to improvements in fatigue resistance, dimensional accuracy, and crack healing [24]. They are also useful for treating components used in environments where water contamination must be avoided, such as sensors and electronic parts in aerospace applications, where moisture exposure can be detrimental [25,26,27].

However, solid confinements must be carefully tailored to match complex part geometries. Even surface irregularities, such as high roughness, can hinder their effectiveness [28]. To overcome these issues, gel-like confinements have gained interest as they potentially combine the benefits of both water and solid confinements.

In this study, the effects of three types of gel-like confinements were investigated and compared to water in the LSP treatment of a nickel-based alloy, with the aim of encouraging further research in this promising field.

## 2. Materials and Methods

The LSP process was conducted on In-718 specimens, with chemical composition tested on a MicroLab 150 (G.N.R., Milan, Italy), presented in Table 1. The material was rolled and stress-relieved at 1065 °C for 90 min by using an MP-3 (Grejac Komerc, Becej, Serbia) laboratory oven with Ar atmosphere. This procedure is in full accordance with the ASTM F3055 standard [29].

**Figure 2:**
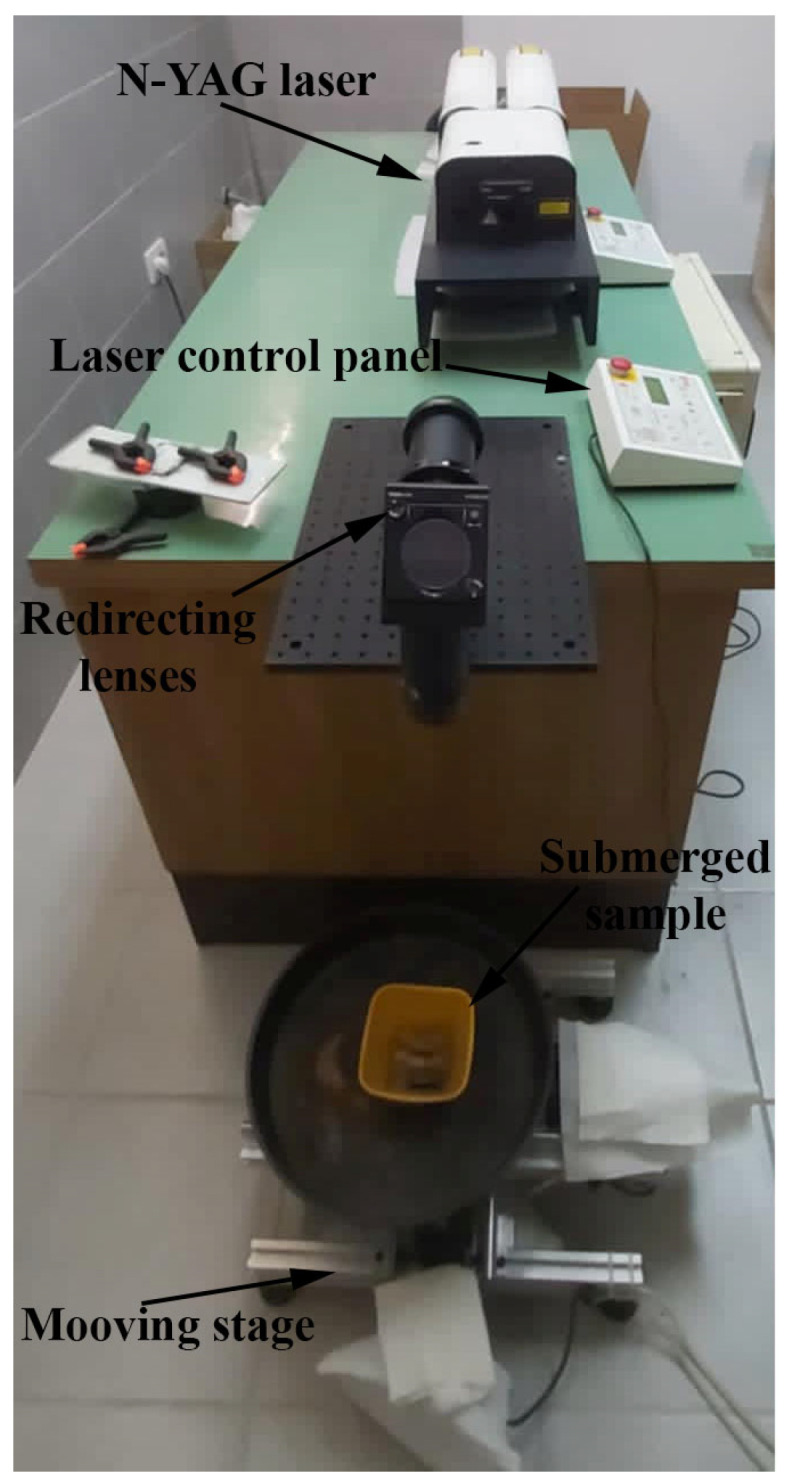
Experimental setup.

The LSP process was performed on In-718 plates, with dimensions of 100 × 50 mm and the thickness of 6.86 mm. The LSP process was performed by using a Twins (Quantel, Lannion, France) Nd:YAG 10 ns pulsed laser (Figure 2). Two wavelengths were used: 532 and 1064 nm, with a spot size of Ø1 mm. A ½ overlapping pattern was applied to form the treated area of 12 × 12 mm, which was chosen in accordance with [30]. LSP was performed with 0.47 J energy in seven passes, which was determined as optimal from this group’s previous experiences and publications [31]. Each set of parameters was used to form two treated squares, of which one was used for residual stress measurement, while the other was used for microstructural and microhardness testing.

Laser treatment was performed in four confinements, with distilled water as a control specimen, and three gel-like confinements: Sikaflex Crystal Gel (Sika AG, Baar, Switzerland), Neutro Gel (ultrasound gel; Daplex, Belgrade, Serbia), and Vaseline (Medical Pharm, Zemun, Serbia). To improve the readability of the material, a designation system was devised, as shown in Table 2.

Microstructural analysis and microhardness testing were performed on specimens initially cut using an SP43 (Makino, Tokyo, Japan) wire electrical discharge machining (EDM) system, equipped with a 0.25 mm Megacut wire (Bedra, Heuchelheim, Germany). Metallographic preparation was conducted in a Struers laboratory, starting with mounting, followed by grinding with SiC abrasive papers (grit sizes ranging from P500 to P2500) and polishing using diamond suspensions with particle sizes of 6 µm, 3 µm, 1 µm, and ¼ µm. Final surface etching was carried out using aqua regia (a mixture of HNO_3_ and 3 parts HCl).

Residual stress measurements were conducted using a hole-drilling method, with a Prism (Stresstech, Vaajakoski, Finland) device at multiple positions and a rotation speed of 40,000 rpm, starting at the surface and proceeding in 0.05 mm increments to a maximum depth of 0.8 mm. Each measurement was repeated three times, and the average values were reported.

Microhardness testing was carried out using a Tukon 1102 (Buehler-Wilson, Uzwill, Switzerland) hardness tester, applying a 50 g load. Measurements were taken at 0.05 mm intervals to a depth of 0.8 mm. As with residual stress measurements, three readings were taken and averaged to obtain the final values.

## 3. Results

### 3.1. Residual Stresses

**Figure 3:**
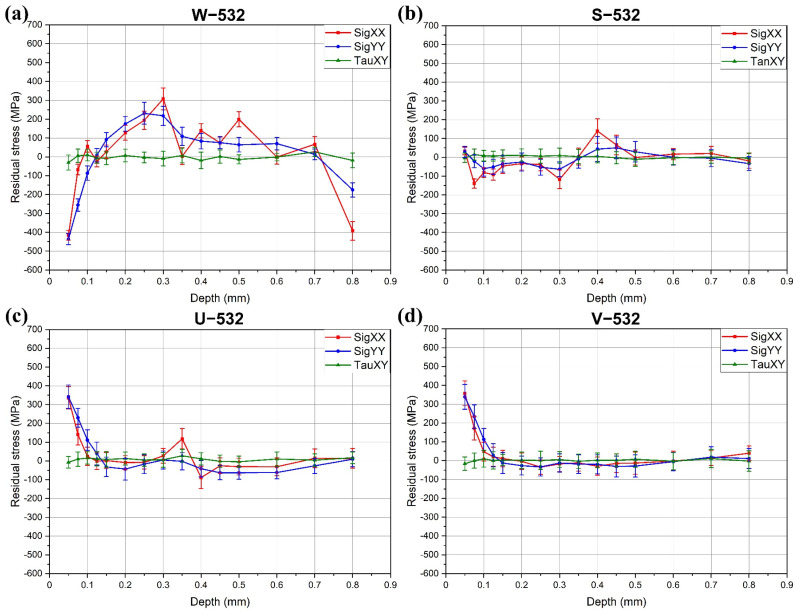
Residual stresses in the specimens treated with 532 nm: (**a**) W−532; (**b**) S−532; (**c**) U−532; (**d**) V−532.

**Figure 4:**
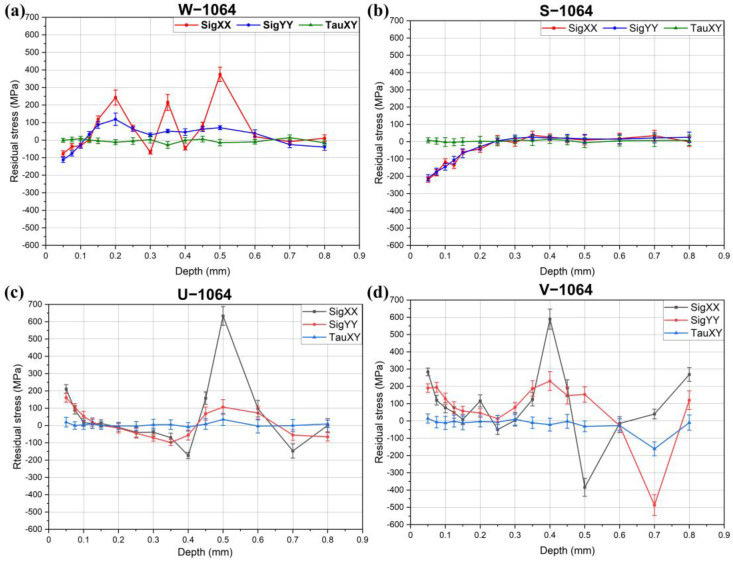
Residual stresses in the specimens treated with 1064 nm: (**a**) W-1064; (**b**) S-1064; (**c**) U-1064; (**d**) V-1064.

The results of the residual stress measurements are presented in Figure 3 and Figure 4. Figure 3 shows the results of LSP treatment using a 532 nm wavelength. These results indicate that water confinement (Figure 3a) induces the highest compressive residual stress at the material surface, exceeding −400 MPa. Compressive stresses extended to a depth of approximately 0.15 mm, beyond which they transitioned into tensile stresses. These tensile values gradually approached the residual stress level of the untreated base material, likely due to the annealing effect from prior thermal processing.

Alternative confinement methods were less effective in inducing compressive stresses. However, ultrasonic gel and Vaseline confinements produced stable and consistent residual stress profiles, with relatively smooth curves and minimal fluctuations. Notably, shear stresses (τ) were largely unaffected by the LSP process, remaining close to 0 MPa throughout the measured depth.

The residual stress results for the specimens treated with a 1064 nm wavelength are presented in Figure 4. Similar to the results shown in Figure 3 for the 532 nm treatment, ultrasonic gel and Vaseline did not provide sufficient confinement to significantly enhance plasma-induced stress waves. Water confinement was partially effective, inducing compressive stresses of approximately −100 MPa. However, notable fluctuations were observed in the residual stress profile between 0.15 mm and 0.6 mm, including distinct tensile stress peaks at 0.2 mm, 0.35 mm, and 0.5 mm.

The most effective confinement in this case was crystal gel, which generated the highest compressive residual stresses, exceeding −200 MPa. The compressive stress region decreased to 0 MPa at a depth of 0.2 mm, beyond which the residual stress gradually returned to the baseline level of the base material (0 to 40 MPa)—a result of prior annealing.

As with the specimens treated at 532 nm, shear stresses (τ) remained largely unaffected by the LSP process and stayed near neutral values across all the specimens.

### 3.2. Microhardness Values

**Figure 5:**
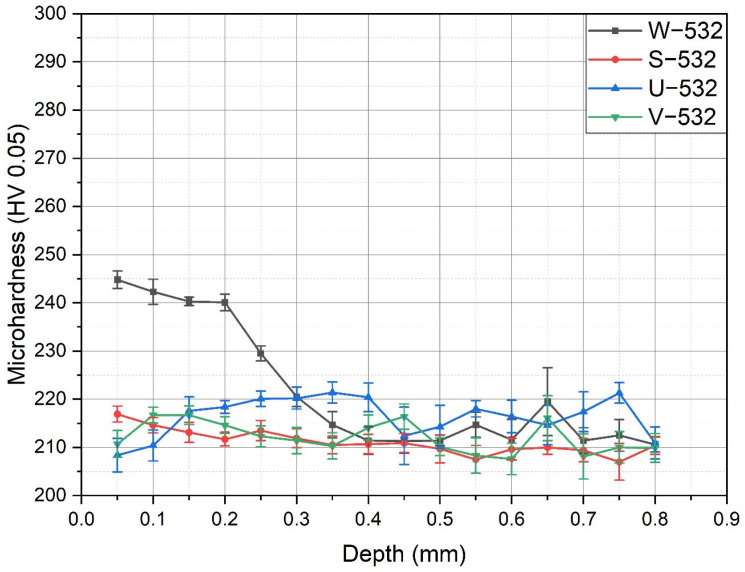
Microhardness values in the specimens treated with 532 nm.

**Figure 6:**
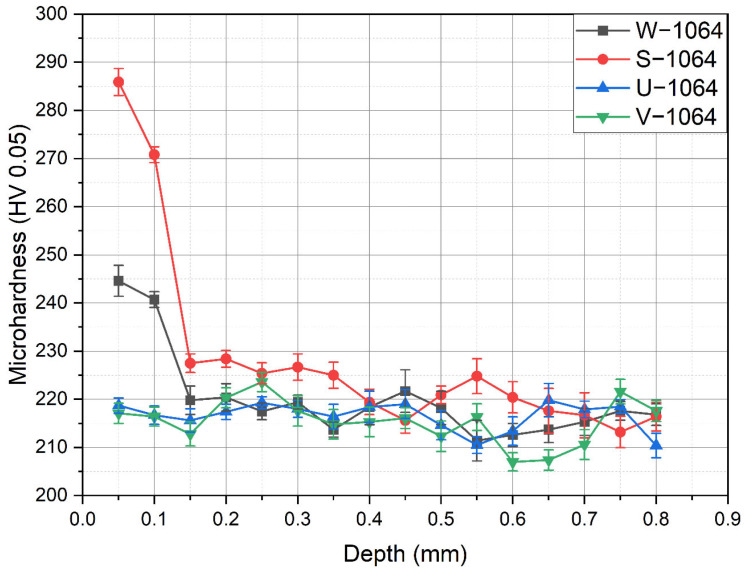
Microhardness values in the specimens treated with 1064 nm.

The microhardness values of the LSP-treated specimens with different confinements are presented in Figure 5 and Figure 6. Figure 5 shows the microhardness results obtained using a 50 g load (HV_0.05_) for the specimens treated with a 532 nm laser and various confinement types. It is clear that water confinement (W−532) provides the highest surface microhardness, exceeding 240 HV_0.05_. At greater depths, the microhardness gradually decreased, stabilizing around 210–220 HV_0.05_. In contrast, the specimens with other confinements showed either a slight increase in hardness near the surface (S−532) or a decrease in microhardness (U−532 and V−532). In all these cases, the microhardness of the base material remained unchanged, similar to that of the W−532 specimens, indicating that the base material was not significantly affected by the treatment.

The microhardness results for the specimens treated with a 1064 nm wavelength are shown in Figure 6. Similar to the findings with the 532 nm wavelength, the base microhardness of specimens W−1064 and S−1064, as well as U−1064 and V−1064, remained between approximately 210 and 220 HV_0.05_ across the measured depth range, indicating no significant change beyond the LSP-affected surface.

Specimens W−1064 and S−1064 exhibited a marked increase in microhardness near the surface, where the LSP effect was most pronounced. In specimen W−1064, the maximum microhardness reached the range of 240–250 HV_0.05_, similar to W−532; however, the depth at which this elevated hardness was measured was 0.1 mm, compared to 0.2 mm in W−532. The highest microhardness was observed in specimen S−1064, where the maximum value approached approximately 300 HV_0.05_, showing the most significant effect of the LSP treatment.

### 3.3. Microstructure

**Figure 7:**
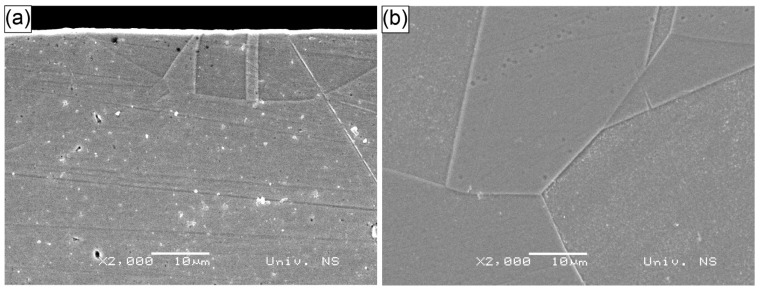
SEM micrograph of specimen W−532: (**a**) surface; (**b**) center.

**Figure 8:**
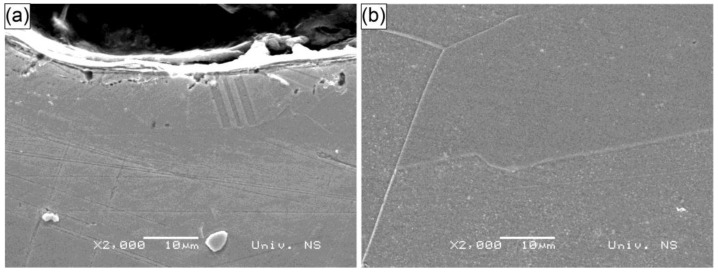
SEM micrograph of specimen S−532: (**a**) surface; (**b**) center.

**Figure 9:**
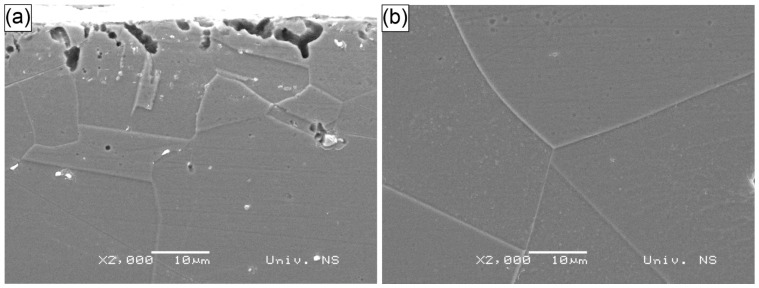
SEM micrograph of specimen S−1064: (**a**) surface; (**b**) center.

The microstructures of representative specimens processed with water and Sikaflex confinements, treated with 532 nm and 1064 nm wavelength LSP, are shown in Figure 7, Figure 8 and Figure 9. Figure 7 presents the W-532 specimen, where the grain size at the center of the specimen was significantly larger than at the surface, measuring approximately 100 µm. This larger grain size was likely a result of the annealing process conducted prior to the LSP treatment. In contrast, the surface grain size was much smaller, around 10 µm, indicating grain refinement due to the LSP process. These smaller grains appear to be a result of twinning, which is often induced by LSP. The grain refinement extended to a depth of 10–25 µm, as shown in Figure 7, Figure 8 and Figure 9.

The microstructures of the S−532 specimen, showing both the surface and central areas, are presented in Figure 8. Similar to the W−532 specimen, the grains in the center of the S−532 specimen were significantly larger than those at the surface, with grain sizes around 100 µm. However, unlike W−532, the twinning mechanism was more pronounced at the surface of the S−532 specimen, occurring at a slightly shallower depth but resulting in multiple, finer twins. These twins were a consequence of plastic deformation induced by the LSP process.

Despite the shallower depth of the affected region, the surface area showing the twinning was smaller, narrower, and more irregular. Additionally, the surface of the S−532 specimen exhibited pits and damage, which were attributed to the aqua regia etching process rather than the LSP treatment. These surface imperfections were caused by the etchant leaking from the small gap between the specimen and the mounting plastic interface.

The microstructure of the S−1064 specimen, shown in Figure 9, exhibited similar behavior to the previously analyzed specimens, W−532 and S−532. The base material microstructure, depicted in Figure 9b, showed a grain size comparable to that observed in the other specimens. At the surface, refined grains were visible, some of which were likely the result of the twinning process, forming a relatively well-defined surface layer. As with the S−532 specimen, large pits were present on the surface, which were attributed to the effects of the aqua regia etching process during metallographic preparation.

## 4. Discussion

In this study, the LSP process was applied to an annealed In-718 base material using various confinements, including water as the traditional material and three types of gel-like materials, at two commonly used wavelengths, 532 nm and 1064 nm. In addition to residual stress measurements, microhardness and microstructures were examined both at the surface and in the central section of the specimens.

Water confinement proved to be effective when used in conjunction with both wavelengths used, 532 and 1064 nm. A higher compressive residual stress was obtained with 532 nm, which aligns with findings reported in [32]. This is confirmed by the results of microhardness, where a higher rise was obtained with the 532 nm wavelength. This result is in accordance with microstructures, which show considerable refinement in the surface layer compared to the central section. The refinement observable on the SEM, reached the maximum of 25 µm, which is comparable to the value of 20–30 µm obtained by Zhang et al. [33] who applied the LSP process to a AW7075-T6 alloy and higher than in the work by Li et al. [34], who obtained 1 µm in a K417 nickel-based alloy. The results show that the average grain size on the peened surface of the specimen was refined to 20–30 μm after LSP, and a residual compressive stress layer with a thickness of approximately 0.5 mm was induced on the surface.

The second confinement type, Sikaflex, proved ineffective when applied with the 532 nm wavelength, resulting in only a moderate increase in microhardness near the surface. However, there was some partial grain refinement at the surface, which, similar to the effect with water confinement, occurred in the form of twinning. When applied with the 1064 nm wavelength, clear compressive stresses appeared at the surface. These stresses, however, were lower than those achieved with water confinement at 532 nm, but higher than those obtained with water confinement at 1064 nm. The microhardness was the highest among all the specimens tested, reaching nearly 300 HV_0.05_, but the increased microhardness was observed at a shallower depth compared to the W-532 specimen.

Le Bras et al. [32] also demonstrated the successful application of the transparent acrylate-based polymer tape; however, gels can provide better coverage of the surface profile and are quicker to be applied in industrial conditions.

Finally, when considering the last two gel types—ultrasonic gel and Vaseline—they were found to be ineffective in inducing compressive residual stress or in significantly increasing surface microhardness. This is the result of their lower structural integrity compared to Sikaflex. In addition, the harder Sikaflex (48 Shore A [35]) behaves similarly to typical adhesive tapes with the hardness range of 40 to 95 Shore A [36] and is closer to the polymer and glass, rendering the wavelength of 1064 nm more effective compared to the 532 nm wavelength. Furthermore, Sikaflex crystal gel is adhesive as well, which gives an additional contribution to this gel’s superiority compared to the other two tested gels. That is particularly important when higher-surface-roughness specimens are LSP-treated, such as additively manufactured specimens in industrial applications with Ra = 8–13.6 µm, which is considerably higher compared to milled specimens (Ra = 0.23–0.78 µm) [37].

Upon analyzing the microstructure, it can be concluded that the grain refinement, compared to the relatively coarse grain structure resulting from the annealing process applied prior to LSP, is closely related to the formation of twins. Twinning, which results in a mirror-image microstructure, occurs when atoms slide past each other due to localized plastic deformation caused by the high-pressure pulse effects, or the mechanical shock waves from the high-pressure plasma [38].

Twinning typically occurs when the applied stress is not aligned with the slip direction or when the material is subjected to high strain rates—both of which apply to the LSP process. Additionally, twinning is more common in materials with specific crystal structures, such as face-centered cubic (FCC) or hexagonal close-packed (HCP) [39,40]. Since Inconel alloys have a typical FCC crystal structure, combined with high strain rates and localized deformation, twinning becomes an important additional deformation mechanism. This contributes to the material’s improved mechanical properties, such as the enhanced microhardness observed in this study, as well as potentially increased resistance to crack initiation, leading to improved fatigue strength [41].

## 5. Conclusions

Based on the results presented and considering the limitations of this study, the following conclusions can be drawn:

- Compressive residual stress can be achieved by LSP combined with gel confinements. To maximize the effectiveness of gels as a confinement for LSP, a wavelength of 1064 nm is preferred over 532 nm.
- The effectiveness of the optimal gel at 1064 nm is lower in terms of inducing compressive residual stresses compared to water confinement at 532 nm. However, at 1064 nm, gel confinement is more effective than water.
- When considering gel-like confinements, special attention should be given to their adhesive properties and structural integrity to ensure they are not significantly affected by the laser or the interaction with the specimen surface. This is particularly important for cast, welded, or additively manufactured specimens, in addition to rolled specimens.
- Microhardness follows the trends of residual stress, particularly in terms of the depth of increased microhardness, rather than the maximal microhardness value. Water confinement at 532 nm resulted in a greater depth of increased microhardness compared to the optimal gel at 1064 nm. Microhardness values at greater depths were similar across all specimens, reflecting the annealing process conducted prior to the LSP treatment.
- The primary mechanisms for increasing surface microhardness are grain refinement and twinning in the surface layer. This is facilitated by the FCC structure of the base material and the high strain rate of the LSP process.

Therefore, gels can serve as a viable alternative to water, although they are less effective both in inducing compressive stresses and achieving depth compared to water, particularly for applications sensitive to water due to the presence of sensors or electronics. Future work will encompass a deeper analysis of other types of adhesive transparent gels, as well as the influence of LSP on fatigue and corrosion, supported by EBSD (electron backscatter diffraction).

## Figures and Tables

**Table 1:** Parent metal chemical composition in wt.%, Ni balance.

C	Mn	Si	P	S	Cr	Co	Mo	Nb	Ta	Ti	Al	Cu	B
0.025	0.05	0.06	0.006	0.005	17.96	0.5	3.20	5.36	0.007	0.95	0.56	0.02	0.005

**Table 2:** Designation system.

Wavelength	Confinement			
	Water	Sikaflex Crystal Gel	Ultrasound Gel	Vaseline
532 nm	W−532	S−532	U−532	V−532
1064 nm	W−1064	S−1064	U−1064	V−1064

## Data Availability

The original contributions presented in this study are included in the article. Further inquiries can be directed to the corresponding author.
